# Adiposity in childhood brain tumors: A report from the Canadian Study of Determinants of Endometabolic Health in Children (CanDECIDE Study)

**DOI:** 10.1038/srep45078

**Published:** 2017-03-22

**Authors:** Kuan-Wen Wang, Russell J. de Souza, Adam Fleming, Sheila K. Singh, Donna L. Johnston, Shayna M. Zelcer, Shahrad Rod Rassekh, Sarah Burrow, Katrin Scheinemann, Lehana Thabane, M. Constantine Samaan

**Affiliations:** 1 Medical Sciences Program, McMaster University, Hamilton, Ontario, Canada; 2 Department of Pediatrics, McMaster University, Hamilton, Ontario, Canada; 3 Division of Pediatric Endocrinology, McMaster Children’s Hospital, Hamilton, Ontario, Canada; 4 Department of Health Research Methods, Evidence and Impact, McMaster University, Hamilton, Ontario, Canada; 5 Division of Pediatric Hematology/Oncology, McMaster Children’s Hospital, Hamilton, Ontario, Canada; 6 Division of Neurosurgery, Department of Surgery, McMaster Children’s Hospital, Hamilton, Ontario, Canada; 7 McMaster Stem Cell and Cancer Research Institute, McMaster University, Hamilton, Ontario, Canada; 8 Division of Pediatric Hematology/Oncology, Children’s Hospital of Eastern Ontario, Ottawa, Ontario, Canada; 9 Pediatric Hematology Oncology, London Health Sciences Center, London, Ontario, Canada; 10 Division of Pediatric Hematology/Oncology/BMT, Department of Pediatrics, British Columbia Children’s Hospital, Vancouver, BC, Canada; 11 Division of Orthopedic Surgery, Department of Surgery, McMaster University Medical Centre, Hamilton, Ontario, Canada; 12 Division of Hematology/Oncology, University Children’s Hospital, Basel, Switzerland; 13 Department of Anesthesia, McMaster University, Hamilton, Ontario, Canada; 14 Centre for Evaluation of Medicines, St. Joseph’s Health Care, Hamilton, ON, Canada; 15 Biostatistics Unit, St. Joseph’s Healthcare-Hamilton, Ontario, Canada

## Abstract

Children with brain tumors (CBT) are at high risk of cardiovascular diseases and type 2 diabetes compared to the general population. Recently, adiposity has been reported to be more informative for cardiometabolic risk stratification than body mass index (BMI) in the general population. The goal of this study is to describe the adiposity phenotype in CBT, and to establish adiposity determinants. We recruited CBT (n = 56) and non-cancer controls (n = 106). Percent body fat (%FM), waist-to-hip ratio (WHR) and waist-to-height ratio (WHtR) were measured to determine total and central adiposity, respectively. Regression analyses were used to evaluate adiposity determinants. CBT had higher total and central adiposity compared to non-cancer controls despite having similar BMI measurements. Those with tumors at the supratentorial region had increased total and central adiposity, while those who received radiotherapy had increased total adiposity. In conclusion, CBT have increased total and central adiposity in the presence of similar BMI levels when compared to non-cancer controls. Adiposity, especially central adiposity, is a potential cardiometabolic risk factor present relatively early in life in CBT. Defining interventions to target adiposity may improve long-term outcomes by preventing cardiometabolic disorders in CBT.

Brain tumors are the most common pediatric solid tumors^1^. Groundbreaking discoveries in tumor biology and advances in diagnosis and therapy have significantly improved the survival of many of these children^2^. As the number of survivors has risen, it has become evident that this group is at risk of developing chronic morbidities^3^,^4^ and premature mortality^5^,^6^.

Recent evidence suggests that adult survivors of childhood brain tumors are at risk of cardiovascular diseases, including stroke, cardiac events, and type 2 diabetes^7^,^8^,^9^,^10^. As obesity is an independent risk factor for cardiometabolic disorders in the general population, it may provide an explanation of the added cardiometabolic risk in survivors^11^. However, when obesity rates are measured by using Body Mass Index (BMI), children with brain tumors (CBT) are reported to have BMI levels that are either close to or slightly higher than rates in the general population, which does not seem to explain this increased cardiometabolic risk in survivors^12^,^13^.

While BMI is the most widely used clinical measure of obesity, it does not distinguish the relative contribution of fat, muscle, or bone to body mass, which are considerably variable in growing children^14^.

On the other hand, adiposity may be a better measurement to determine cardiometabolic risk in CBT. Adiposity is defined as the presence of fat in and outside the adipose tissue, including muscle and hepatic fat depots. The adipose depot is composed of a subcutaneous compartment, which is considered protective against cardiometabolic risk^15^,^16^. On the other hand, the visceral adipose compartment secretes inflammatory cytokines which can lead to insulin resistance, and is linked to adverse cardiometabolic outcomes^17^.

Measures of total adiposity (fat mass percentage; %FM) and central adiposity, including waist-to-hip ratio (WHR) and waist-to-height ratio (WHtR), have been shown to be more robust predictors of cardiometabolic health and risk compared to BMI^18^,^19^,^20^,^21^,^22^,^23^,^24^, with WHtR emerging as a strong indicator of intra-abdominal fat^25^.

However, adiposity is not routinely measured in children, including pediatric cancer patients. While brain tumors are a heterogeneous group, a common tumor classically reported to be associated with obesity is craniopharyngioma^26^. There have been very few reports on the evaluation of obesity in other brain tumor subtypes and beyond hypothalamic obesity^27^,^28^. As BMI-based obesity rates are similar between CBT and controls yet CBT have high risk of cardiometabolic disorders, we hypothesized that CBT, excluding craniopharyngioma, have higher adiposity when compared to non-cancer controls. This excess adiposity may contribute to adverse cardiometabolic outcomes and premature mortality. A secondary aim of this study was to investigate the determinants of adiposity in CBT.

## Results

We included 56 CBT (n = 23 female) and 106 non-cancer controls (n = 51 female) in this study. The characteristics of the study population are reported in Table 1.

The two groups were similar in terms of age (CBT: 5.20–42.70 years; controls: 5.40–18.80 years; p-value 0.59) and sex distribution (p-value 0.39). The CBT group had more participants in prepubertal stage (n = 19, 33.90%) versus controls (n = 16, 15.10%). Age of diagnosis of brain tumor was 9.10 ± 4.90 years, and average time since diagnosis was 5.60 ± 5.10 years.

As reported previously^29^, CBT were shorter (150.60 ± 25.20 versus 161.70 ± 15.30 cm, p-value = 0.002) and weighed less (52.40 ± 24.10 versus 59.00 ± 20.80 kg, p-value = 0.02) than the control group.

The %FM correlated with central adiposity (Spearman’s rho test WHR 0.31, p-value < 0.001; WHtR 0.73, p-value < 0.001). Central adiposity measures were highly correlated with each other as well (Spearman’s rho test 0.67, p-value < 0.001).

The total screen time and sleep duration were similar between the two groups (Table 1). The most common tumor subtypes in participants included gliomas (n = 34, 60.70%) and Primitive Neuroectodermal tumors (PNET)/medulloblastoma (n = 11, 19.60%) (Table 2). The tumors were distributed between supratentorial (n = 26, 46.40%) and infratentorial regions (n = 30, 53.60%) (Table 2), with only 7 patients (12.50%) having tumors involving the hypothalamus. The therapeutic modalities were used in the management of brain tumors are shown in Table 2. Surgery alone was the most common treatment modality (n = 18, 32.10%), followed by a combination of surgery, chemotherapy and radiotherapy (n = 15, 26.80%). Chemotherapy alone was noted in five cases (8.90%), and radiotherapy alone was implemented in one patient (1.80%). Four patients (7.10%) received surgery and chemotherapy, and four (7.10%) received surgery and radiotherapy; one received radiotherapy and chemotherapy (1.80%).

In the 22 participants who received radiotherapy, the radiotherapy dosage was 47.10 ± 12.40 Gy. Sixteen participants received craniospinal irradiation (72.70%), and six received cranial irradiation (27.30%). Eight patients were being managed with watch-and-wait strategy (14.30%).

Post-therapy endocrinopathies were observed in 14 (26.80%) CBT participants. Among this group, a single diagnosis was made in seven patients including hypothyroidism (n = 3, 21.40%), growth hormone deficiency (n = 2, 14.30%), hypogonadism (n = 1, 7.10%), and precocious puberty (n = 1, 7.10%). The other seven patients had multiple hormonal deficiencies including hypothyroidism (n = 5, 35.70%), growth hormone deficiency (n = 6, 42.90%), hypogonadism (n = 4, 28.60%), adrenocorticotropic hormone deficiency (n = 4, 28.60%), diabetes insipidus (n = 3, 21.40%), and precocious puberty (n = 1, 7.10%). All endocrinopathies were treated appropriately.

### Adiposity patterns in CBT and controls

To determine if CBT have enhanced adiposity compared to non-cancer controls, we used logistic regression analysis.

CBT had higher total adiposity compared to controls (%FM 25.50 ± 9.60% versus 22.40 ± 9.30%; β = 1.51, 95% CI = 1.08, 2.10, p-value = 0.016). CBT also had higher central adiposity compared to controls including higher WHR (0.87 ± 0.07 versus 0.82 ± 0.09; β = 7.53, 95% CI = 2.30, 24.64, p-value = 0.001) and a trend of higher WHtR (0.47 ± 0.06 versus 0.45 ± 0.08; β = 0.34, 95% CI = 0.12, 1.02, p-value = 0.053).

Importantly, there were no differences in BMI and overweight/obesity rates between CBT and non-cancer controls (Table 1). BMI correlated with total adiposity (%FM) in CBT and controls (Spearman’s rho test CBT 0.50, p-value < 0.001; controls 0.76, p-value < 0.001). BMI also correlated with WHR in controls but not in CBT (Spearman’s rho test CBT 0.41, p-value 0.12; controls 0.17, p-value 0.038). Furthermore, BMI correlated with WHtR in CBT and controls (Spearman’s rho test CBT 0.51, p-value < 0.001; controls 0.73, p-value < 0.001). These results demonstrate that CBT have higher total and central adiposity compared to non-cancer controls, in the presence of similar obesity rates based on BMI measurements.

### Determinants of adiposity in survivors and controls

To define the determinants of adiposity, we conducted separate exploratory subgroup analyses using multivariate linear regression for CBT and controls (Table 3 for CBT; Supplementary Table S1 for controls). Dietary data are included in Table 4.

Females in the control group had higher total adiposity, while males had increased WHR, and puberty was associated with all measures of adiposity. These trends were not noted in CBT.

CBT with Supratentorial tumors had increased total adiposity (β −1.83, SE 0.80, p-value 0.028), with trended association with central adiposity (WHR β −0.37, SE 0.21, p-value 0.08; WHtR β −0.53, SE 0.27, p-value 0.06) (Table 3).

CBT who received radiotherapy had higher %FM (β = 1.65, SE 0.79, p-value = 0.046). However, radiotherapy type (craniospinal versus cranial irradiation) and radiation dose did not correlate with %FM (Spearman’s rho test radiotherapy type r 0.13, p-value 0.57; Dose r 0.24, p-value 0.36), WHR (Spearman’s rho test radiotherapy type r 0.18, p-value 0.43; Dose r 0.33, p-value 0.17), or WHtR (Spearman’s rho test radiotherapy type r 0.10, p-value 0.67; Dose r 0.24, p-value 0.3)’.

While 27 (48.2%) CBT were treated with corticosteroids, there was no association between steroid use and %FM (β = 0.68, SE 0.62, p-value = 0.28), WHR (β = 0.04, SE 0.19, p-value = 0.81), or WHtR (β = 0.21, SE 0.21, p-value = 0.32) (Table 3).

When examining the contribution of lifestyle factors (diet, physical activity, screen time, sleep duration) to adiposity in controls, physical inactivity trended with WHR, while screen time was associated with WHtR. Diet and sleep duration were not associated with adiposity measures. None of the lifestyle factors were associated with total or central adiposity measures in CBT (Table 3 for CBT; Supplementary Table S1 for controls; Diet data Table 4).

## Discussion

The improved survival rates of children with brain tumors have been hindered by premature mortality and the development of morbidities. Of particular importance, recent evidence confirms that survivors are at risk of type 2 diabetes and cardiovascular diseases^7^,^8^,^9^,^10^. In this study, we demonstrate that adiposity, one of the most important determinants of cardiometabolic risk, is enhanced in CBT when compared to non-cancer controls.

Importantly, the adipose phenotype noted in CBT is evident with equivalent overweight/obesity rates to controls based on BMI measurements.

It has been reported that BMI can underestimate the prevalence of obesity in childhood cancer survivors, including survivors of brain tumors^18^. Until further knowledge is generated of the potential role of early excess adiposity in programming future cardiometabolic risk in CBT, there is a need to measure both BMI and adipose depots, and to continue to attempt to define their determinants. Our data are consistent with studies that used dual X-ray absorptiometry (DXA) scans^30^, and reported the presence of higher total adiposity in cancer survivors who were treated with cranial irradiation^30^. The first study identified impaired mobility as an association of adiposity; the second study recruited patients with different cancers including brain tumors, and used siblings as a control group. The latter study identified male sex and screen time as risk factors of adiposity^30^. Our study population included CBT exclusively, with non-cancer controls as a comparison group. This may explain why the previously identified risk factors were not associated with adiposity in our study.

An important contribution of our study is that it provides evidence for the use of clinically feasible measures to determine adiposity in CBT. This has important implications for settings where access to DXA is not practical or possible, allowing clinicians to estimate the adiposity patterns in their survivor populations.

Our data also demonstrate that tumor location and radiotherapy have important associations with adiposity. Supratentorial tumors were associated with enhanced total and central adiposity, while radiotherapy was associated with excess total adiposity.

While tumors and their treatment can lead to anatomical or functional hypothalamic-pituitary damage with pituitary hormonal deficiencies^31^, disruption of hypothalamic satiety signaling and reduced basal metabolic rate that can drive obesity^26^, these factors may also contribute to excess adiposity.

Our results did not corroborate previous evidence of the association of higher doses of radiotherapy with obesity in childhood cancers, including brain tumors^32^,^33^. While these studies used BMI to measure obesity, our results suggest that adiposity may be associated with radiotherapy regardless of dosage. Clarifying the effect of radiotherapy type, dosing and fractionation on adiposity is an important question to address in CBT.

Endocrinopathies have been reported to increase the risk of higher BMI, but their effect on adiposity patterns in CBT early on requires further study, as these effects may become more apparent as CBT age. Given that radiation dosage is associated with hormonal abnormalities in cancer survivors, the effect of radiation dosage on adiposity may have been masked in our population who were treated for existing endocrinopathies^34^.

It has been reported that certain tumors including craniopharyngiomas, pilocytic astrocytomas, and medulloblastomas are associated with elevated BMI^33^. We purposefully excluded craniopharyngiomas, to determine the contribution of other tumors to the adipose phenotype in CBT. A larger sample size is needed to clarify whether adiposity is driven by specific tumor types.

Several lifestyle factors are associated with obesity in the general pediatric population, Including excess caloric intake from sugar-sweetened beverages, prolonged screen time, and short sleep duration. Physical inactivity has been a controversial determinant of obesity in children^35^,^36^,^37^,^38^,^39^.

While biological (sex), hormonal (puberty) and lifestyle factors were associated with adiposity in controls, none emerged as an explanation of the enhanced adiposity profile in CBT, which was associated with tumor location and radiotherapy.

The lack of association of diet with adiposity in our study is consistent with a study in craniopharyngioma patients, which revealed that physical inactivity, and not nutritional factors, were associated with higher adiposity^40^. As our study is cross-sectional, one caveat is that the dietary patterns may have changed from the time of diagnosis onwards. Longitudinal studies are needed to clarify the link between diet and adiposity in CBT.

The association of physical inactivity with childhood obesity and its use as a treatment for obesity has yielded inconsistent results^41^,^42^,^43^,^44^. In CBT, physical inactivity can be driven by treatment-related pulmonary and cardiac dysfunction^45^,^46^, reduced muscle strength and fitness^47^, fatigue, sleep disturbance^48^, mental health issues, visual impairment, imbalance and pain^49^,^50^. Further studies on the association of physical inactivity with adiposity, and fat mass modification by targeted interventions in CBT are needed.

Our data suggest that within few years from having a brain tumor, CBT are following the secular lifestyle trends noted in the general population. However, the effect of adopting these trends on adiposity and cardiometabolic risk in CBT can be disproportionate, due to the added burden of the tumor and its treatment. Multipronged, personalized, and sustained interventions are needed in CBT, as adiposity is only one of many risk factors that may respond to lifestyle alteration.

There are several limitations to our study. While the WHR and WHtR demonstrated the presence of excess central adiposity in CBT, it is not clear if this is due to subcutaneous or visceral fat depot expansion. It is also unclear yet if these adiposity patterns will be sustained as CBT age. In addition, due to the cost and logistics involved we did not measure other fat depots including hepatic and intermyocellular fat. Larger sample size and longitudinal studies of the fat depots are needed starting at diagnosis, to elucidate the evolution of the adiposity patterns in CBT.

As the questionnaires were self-administered, the presence of recall bias is possible. However, this is less likely, as the data collected were related to recent lifestyle factors, and the clinical data related to the tumor and its treatment were collected from the medical records.

## Conclusions

In summary, our study reveals that excess total and central adiposity are present in non-craniopharyngioma population of CBT compared to controls. Adiposity, especially central adiposity, is an important cardiometabolic risk marker that appears in CBT within few years of their diagnosis. Tumor location and radiotherapy are important determinants of the noted adipose phenotype in these patients.

There is a need to understand the determinants of adiposity so that new therapies and prevention strategies can be developed to mitigate premature cardiovascular diseases and type 2 diabetes and improve outcomes in CBT.

## Methods

### Participants

The participants in this study were recruited into the Canadian Study of Determinants of Endometabolic Health in Children (CanDECIDE Study). This is a cohort study based at McMaster Children’s Hospital, a tertiary pediatric academic center in Hamilton, Ontario, Canada. The study protocol and feasibility have been published^51^,^52^. The data reported are cross-sectional data collected at recruitment into the study.

We consecutively recruited CBT from the neurooncology clinics, and non-cancer controls were recruited from orthopedic clinics at the hospital and from the community. The orthopedic clinic controls included healthy children who suffered fractures or sprains and were seen for evaluation. Importantly, all study measures were performed after the fractures or sprains have healed, and participants had returned to their usual lifestyle before the injury. The recruitment period lasted from November 2012-March 2016.

We recruited boys and girls, 5 years and older, who were free of infection for 15 days prior to participation in the study, with no history of autoimmune diseases and not receiving immunosuppressive therapy for 15 days prior to inclusion. The exclusion criteria included active infection, autoimmune diseases, pregnancy or inability to provide informed consent.

### Consent

The Hamilton Integrated Research Ethics Board approved this study. Consent forms were signed by parents if the participants were less than 16 years old, or by the participants if they were 16 years or older^53^. Children 7–15 years of age also signed an additional assent form. Informed consent was obtained from all participants. The study was conducted in accordance with appropriate clinical practice guidelines and national legal requirements.

### Sociodemographic and clinical data

Data collected during the initial encounter with potential participants included self-reported age and sex, and this was confirmed from the medical records. Additional data collected from the medical records included age at diagnosis, tumor type, location, details of treatments received, and associated endocrinopathies and their treatment. Pubertal staging was assessed by pictorial Tanner pubertal staging in girls (>8 year old) and boys (>9 year old)^54^.

Height and weight were measured to the nearest one tenth of a centimeter and one tenth of a kilogram using a stadiometer and an electronic weighing scale (Seca, USA), respectively. Body mass index (BMI) was calculated as kg/m^2^. BMI percentile was obtained using the Children’s BMI Tool for Schools^55^ and BMI z-score were determined from the Centers for Disease Control and Prevention (CDC) growth chart^56^. Sitting systolic and diastolic blood pressures were measured twice using the right arm with an automated blood pressure monitor (Welch Allyn, Inc., USA) and the average values of these two measurements are reported.

The two commonly used methods to measure body fat include Dual-energy X-ray absorptiometry (DXA) scan and bioelectrical impedance analysis (BIA)^57^. The latter is less expensive, easier to access and perform than DXA. In this study, we used BIA to measure %FM to determine total adiposity. This method has been validated against DXA scans, and the two measures are highly correlated^57^. While the Tanita body fat monitor (Tanita Corporation, Illinois, USA) is portable, it cannot be used on those 18 years and older. In this case, the InBody520 body composition analyzer (Biospace Co., Ltd, Korea) was used to measure %FM. High correlations were established between the Tanita body fat monitor and the InBody520 body composition analyzer when tested on 5–17 year old children (r = 0.87; p-value = 0.001).

Waist and hip circumferences were measured to the nearest one tenth of a centimeter, using a spring-loaded measuring tape (OHAUS Corporation, Canada)^58^. Central adiposity was determined by calculating the WHR and WHtR^21^.

### Diet

Dietary intake was assessed as we previously reported^52^. Briefly, we used items from the Youth and Adolescent Food Frequency Questionnaire^52^,^59^,^60^. This is a questionnaire developed in a US pediatric cohort, and includes questions about food intake based on average portion sizes of different dietary constituents. The number of servings per day was calculated from the questionnaire by multiplying the frequency of consumption by portion size.

Principal component analysis was used to analyze the dietary patterns in participants. This analysis revealed four dietary patterns including prudent, western, high-protein and refined carbohydrate diets (Table 4). The prudent diet included high intake of fruits and vegetables. The western diet included high intake of fried foods, desserts, baked goods, and refined foods (e.g., chips, snacks, candies). The high-protein diet included high intake of meat and eggs. The refined carbohydrate diet included high intake of white bread and low intake of dark (whole grain) bread.

### Physical activity

Physical activity was measured using the Habitual Activity Estimation Scale (HAES)^61^. The participants were asked to indicate their overall physical activity level as very inactive, inactive, somewhat inactive, somewhat active, active, or very active. This data were used to report physical activity levels. The levels were dichotomized into active and inactive for statistical analyses.

### Sleep

Sleep duration (hours/day) was calculated from the difference between the self-reported time the participant went to bed and woke up the next morning. Sleep duration calculated with this method has been shown to correlate well with objective sleep quantification methods^62^.

### Screen time

Total screen time (hours/day) was calculated from the sum of self-reported time spent watching television, using cell phone, computer, computer games, and tablets.

### Statistical analysis

All analyses were performed with SPSS version 20 software^63^. Kolmogorov-Smirnov test was used to test for normality, and variables with non-normal distribution were log-transformed. Age log-transformation revealed no outliers.

We used variance inflation factor to test for collinearity of variables, and found none that were collinear. Multiple imputations were used to handle missing data.

Continuous variables are reported as mean ± SD, and categorical variables are reported as counts (%). Chi-square tests and independent sample t-tests were used to compare brain tumor survivors and controls for categorical and continuous variables, respectively. We used Spearman’s test to assess the correlation of adiposity measures with BMI and with each other in this study.

To assess the association of adiposity with brain tumor status, we used binary logistic regression. The dependent variable (event) was the cancer case status, with 56 events included in the analysis and 106 controls (non-events). Age, sex, %FM, WHR, and WHtR were included as the predictor variables in the analysis. We rescaled the WHR and WHtR coefficients by multiplying the log-transformed data by 10^64^,^65^. Logistic regression was conducted based on the assumption that ten events per predictor variable are needed for the analysis. As there are five predictor variables included in the analysis, our study is sufficiently powered to answer the main study question.

To explore the determinants of the adiposity patterns in CBT and controls, we performed exploratory subgroup analyses of the cancer cases and controls separately with multivariate linear regression analysis. The dependent variables included %FM, WHR, and WHtR.

The predictor variables of interest in CBT included age, sex, puberty, brain tumor histopathology, tumor location, and treatments including surgery, radiotherapy, chemotherapy, and steroids. In addition, we included lifestyle factors encompassing diet, physical activity, screen time, and sleep duration in the analysis. For controls, we included age, sex, puberty, diet, physical activity, screen time, and sleep duration in the analysis. The sample size of 56 events and 106 non-events provide adequate power for this analysis, as two events per variable are required in linear regression analyses to address the question of adiposity determinants in CBT and controls^66^. To analyze the dietary patterns in participants, we used principal component analysis. Twenty-two food items were included in the factor analysis. The number of dietary patterns retained was determined by visual inspection of scree plots in conjunction with eigenvalues (>1.0) and principal component interpretability. The factors were orthogonally transformed by using the varimax rotation to ensure the independence of factors in the structure. Dietary patterns were characterized based on dietary items with their factor loadings ≥|0.30|. The PCA scores for each pattern obtained for each individual represented how closely their food choices reflected one of the empirically-derived dietary patterns, with higher scores reflecting a greater degree of adherence to that dietary pattern^67^.

## Additional Information

**How to cite this article:** Wang, K.-W. *et al*. Adiposity in childhood brain tumors: A report from the Canadian Study of Determinants of Endometabolic Health in Children (CanDECIDE Study). *Sci. Rep.* **7**, 45078; doi: 10.1038/srep45078 (2017).

**Publisher's note:** Springer Nature remains neutral with regard to jurisdictional claims in published maps and institutional affiliations.

## Supplementary Material

Supplementary Information

## Figures and Tables

**Table 1:** Characteristics of study population.

Variables	Non-cancer controls (n = 106)			CBT (n = 56)		
	Total Mean (SD)	Male Mean (SD)	Female Mean (SD)	Total Mean (SD)	Male Mean (SD)	Female Mean (SD)
Age (years)	14.00 (2.80)	14.00 (2.60)	14.00 (3.00)	14.70 (7.10)	14.80 (5.50)	14.50 (9.00)
Sex, No. (%)						
Female	51.00 (48.10)	—	—	23.00 (41.10)	—	—
Male	55.00 (51.90)	—	—	33.00 (58.90)	—	—
Height (cm)	161.70 (15.30)	166.00 (16.80)	157.20 (11.90)	150.60 (25.20)	155.90 (26.10)	143.00 (22.30)
Weight (kg)	59.00 (20.80)	64.30 (25.00)	53.50 (13.30)	52.40 (24.10)	55.20 (23.00)	48.50 (25.50)
BMI (kg/m2)	22.10 (5.60)	22.80 (6.60)	21.40 (4.10)	21.60 (5.50)	21.40 (4.40)	21.80 (6.80)
BMI z-score	0.49 (1.16)	0.58 (1.27)	0.41 (1.02)	0.41 (1.15)	0.32 (1.26)	0.55 (0.96)
BMI category, No. (%)						
BMI%ile < 85	69.00 (65.10)	34.00 (61.80)	35.00 (68.60)	36.00 (64.30)	22.00 (66.70)	14.00 (60.90)
BMI%ile ≥ 85	37.00 (34.90)	21.00 (38.10)	16.00 (31.40)	20.00 (35.70)	11.00 (33.30)	9.00 (39.10)
%FM	22.20 (9.00)	19.10 (9.00)	25.60 (7.80)	25.80 (9.60)	23.00 (9.40)	29.90 (8.60)
WHR	0.82 (0.09)	0.84 (0.08)	0.80 (0.10)	0.87 (0.07)	0.86 (0.07)	0.88 (0.08)
WHtR	0.45 (0.08)	0.45 (0.09)	0.44 (0.07)	0.47 (0.06)	0.47 (0.06)	0.48 (0.07)
Sys BP (mmHg)	107.20 (10.60)	110.40 (10.60)	103.70 (9.60)	104.00 (11.50)	104.10 (11.60)	103.90 (11.80)
Dia BP (mmHg)	67.60 (9.60)	67.10 (10.00)	68.10 (9.10)	66.30 (8.50)	66.20 (8.50)	66.40 (8.80)
Physical activity, No. (%)						
Active	97.00 (91.50)	48.00 (87.30)	49.00 (96.10)	43.00 (76.80)	25.00 (75.80)	18.00 (78.30)
Inactive	9.00 (8.50)	7.00 (12.70)	2.00 (3.90)	13.00 (23.20)	8.00 (24.20)	5.00 (21.70)
Screen time (hours/day)	4.30 (2.60)	4.80 (2.70)	3.80 (2.50)	4.50 (2.70)	4.80 (2.60)	3.90 (2.70)
Sleep duration (hours/day)	9.50 (1.40)	9.70 (1.70)	9.40 (1.10)	9.60 (1.20)	9.40 (1.20)	9.70 (1.10)

Abbreviations: SD, Standard Deviation; BMI, Body Mass Index; %tile, percentile; %FM, fat mass percentage; WHR, waist-to-hip ratio; WHtR, waist-to-height ratio; Sys BP, systolic blood pressure; Dia BP, diastolic blood pressure; mmHg, millimeter Mercury.

**Table 2:** Brain tumor type, location, and treatments.

Variables	No. (%)
**Brain tumor type**	
CNS germ cell tumors	5 (8.90)
PNET/Medulloblastoma	11 (19.60)
Ependymoma	2 (3.60)
Subependymal giant cell astrocytoma	3 (5.40)
Meningioma	1 (1.80)
NF-1, low grade glioma	10 (17.85)
Non-NF-1, low grade glioma	24 (42.85)
**Brain tumor location**	
Supratentorial	26 (46.40)
Infratentorial	30 (53.60)
**Brain tumor treatments**	
Surgery	41 (73.20)
Radiotherapy	22 (39.30)
Chemotherapy	27 (48.20)
No treatment	8 (14.30)
Steroids	27 (48.20)

Abbreviations: PNET, Primitive Neuroectodermal Tumor; NF-1, Neurofibromatosis Type 1.

**Table 3:** The determinants of adiposity in participants with brain tumors.

Variables	%FM		WHR		WHtR	
	β (SE)	P-value	β (SE)	P-value	β (SE)	P-value
Age	−0.12 (0.10)	0.23	−0.28 (0.03)	0.29	−0.02 (0.03)	0.50
Sex	0.55 (0.52)	0.30	−0.13 (0.14)	0.36	−0.13 (0.18)	0.46
Puberty	1.11 (0.99)	0.28	0.28 (0.26)	0.29	0.35 (0.34)	0.32
Brain tumor type	−0.33 (0.44)	0.46	−0.06 (0.11)	0.58	0.08 (0.15)	0.61
Brain tumor location	−1.83 (0.80)	0.028	−0.37 (0.21)	0.08	−0.53 (0.27)	0.06
Surgery	0.91 (0.81)	0.27	0.08 (0.21)	0.69	0.20 (0.28)	0.47
Radiotherapy	1.65 (0.79)	0.046	0.08 (0.21)	0.69	0.22 (0.27)	0.43
Chemotherapy	−0.86 (0.74)	0.25	0.06 (0.19)	0.77	−0.02 (0.25)	0.93
Steroids	0.68 (0.62)	0.28	0.04 (0.16)	0.81	0.21 (0.21)	0.32
Prudent diet	0.13 (0.33)	0.68	0.06 (0.08)	0.44	0.06 (0.11)	0.62
Western diet	0.15 (0.33)	0.64	0.04 (0.09)	0.64	0.17 (0.11)	0.13
High-protein diet	−0.19 (0.27)	0.48	−0.02 (0.07)	0.76	−0.09 (0.09)	0.33
Refined carbohydrate diet	0.38 (0.29)	0.20	0.06 (0.08)	0.43	0.07 (0.10)	0.45
Physical inactivity	−0.89 (0.57)	0.12	−0.12 (0.15)	0.42	−0.26 (0.19)	0.19
Screen time	1.08 (1.33)	0.42	0.14 (0.34)	0.68	0.42 (0.46)	0.37
Sleep duration	6.41 (7.24)	0.38	1.77 (1.87)	0.35	1.44 (2.49)	0.57

Abbreviations: %FM, Percent Fat Mass; WHR, Waist-to-Hip Ratio; WHtR, Waist-to-Height Ratio; SE, standard error.

**Table 4:** Factor loading matrix for dietary patterns in participants.

Items	Factor loadings			
	Prudent	Western	High-protein	Refined carbohydrate
Fruits	**0**.**73**	—	—	—
Vegetables	**0**.**70**	—	—	—
Water	**0**.**57**	—	—	—
Crackers	**0**.**52**	—	—	—
Grain	0.49	—	—	—
Juice	—	−0.18	—	—
Fried Foods	—	**0**.**68**	—	—
Desserts	—	**0**.**61**	—	—
Baked Goods	—	**0**.**56**	—	—
Chips	—	**0**.**53**	—	—
Snacks	—	**0**.**53**	—	—
Candies	—	**0**.**51**	—	—
Poultry	−0.31	—	**0**.**67**	—
Red Meat	—	—	**0**.**63**	—
Eggs	—	—	**0**.**57**	—
Soft Drinks	−0.39	—	0.40	—
Peanut/other nuts	0.30	−0.31	0.38	—
White Bread	—	0.30	—	**0**.**71**
Dark Bread	0.35	—	0.34	**−0**.**67**
Gelatin	—	—	—	**0**.**66**
Fish	—	—	—	0.37
Dairy	—	—	—	0.35
Total variance explained (%)	15.7	10.1	8.4	7.1

Absolute values < 0.30 were not listed in the table, except for juice whose highest value of factor loading is shown.

Absolute values > 0.50 were bolded to emphasize strength of association and determination of dietary patterns.
