# Supplementary material for: Adiposity in childhood brain tumors: A report from the Canadian Study of Determinants of Endometabolic Health in Children (CanDECIDE Study)
Source: Sci Rep. 2017 Mar 22;7:45078. doi: 10.1038/srep45078 (PMC5361156; doi:10.1038/srep45078)
Supplement: Supplementary Information [file srep45078-s1.pdf]

Adiposity in childhood brain tumors: A report from the Canadian Study of Determinants of Endometabolic Health in Children (CanDECIDE Study)

Kuan-Wen Wang[1,2,3], Russell J. de Souza[1,4], Adam Fleming[1,2,5], Sheila K. Singh[6,7], Donna L. Johnston[8], Shayna M. Zelcer[9], Shahrad Rod Rassekh[10], Sarah Burrow[11], Katrin Scheinemann[2,12], Lehana Thabane[1,4,13,14,15], M. Constantine Samaan[1,2,3,4*]
[1]Medical Sciences Program, McMaster University, Hamilton, Ontario, Canada
[2]Department of Pediatrics, McMaster University, Hamilton, Ontario, Canada
[3]Division of Pediatric Endocrinology, McMaster Children's Hospital, Hamilton, Ontario, Canada
[4]Department of Clinical Epidemiology & Biostatistics, McMaster University, Hamilton, Ontario, Canada
[5]Division of Pediatric Hematology/Oncology, McMaster Children's Hospital, Hamilton, Ontario, Canada
[6]Division of Neurosurgery, Department of Surgery, McMaster Children's Hospital, Hamilton, Ontario, Canada
[7]McMaster Stem Cell and Cancer Research Institute, McMaster University, Hamilton, Ontario, Canada
[8]Division of Pediatric Hematology/Oncology, Children's Hospital of Eastern Ontario, Ottawa, Ontario, Canada
[9]Pediatric Hematology Oncology, London Health Sciences Center, London, Ontario, Canada
[10]Division of Pediatric Hematology/Oncology/BMT, Department of Pediatrics, British Columbia's Children's Hospital, Vancouver, BC, Canada
[11]Division of Orthopedic Surgery, Department of Surgery, McMaster University Medical Centre, Hamilton, Ontario, Canada
[12]Division of Hematology/Oncology, University Children's Hospital, Basel, Switzerland
[13]Department of Anesthesia, McMaster University, Hamilton, Ontario, Canada
[14]Centre for Evaluation of Medicines, St. Joseph's Health Care, Hamilton, ON, Canada
[15]Biostatistics Unit, St Joseph's Healthcare-Hamilton, Hamilton, Ontario, Canada

Supplementary Table S1. Factors associated with adiposity patterns in non-cancer controls.

**Variables**	**%FM**		**WHR**		**WHtR**	
	β (SE)	P-value	β (SE)	P-value	β (SE)	P-value
Age	0.14 (0.11)	0.19	0.01 (0.02)	0.59	0.05 (0.04)	0.25
Sex	1.54 (0.41)	<0.001	-0.24 (0.09)	0.01	-0.04 (0.15)	0.78
Puberty	-1.53 (0.78)	0.052	-0.47(0.18)	0.01	-0.68 (0.29)	0.02
Prudent diet	-0.14 (0.20)	0.50	0.07 (0.05)	0.12	-0.02 (0.08)	0.81
Western diet	-0.13 (0.20)	0.50	0.07 (0.04)	0.10	-0.04 (0.07)	0.56
High protein diet	-0.19 (0.20)	0.34	0.007(0.05)	0.88	-0.007 (0.07)	0.93
Refined carbohydrate diet	0.09 (0.20)	0.65	0.01(0.05)	0.80	0.13 (0.07)	0.09
Physical inactivity	0.04 (0.72)	0.95	0.30 (0.16)	0.07	0.10 (0.27)	0.70
Screen time	0.31 (0.80)	0.70	0.31 (0.18)	0.09	0.65 (0.29)	0.03
Sleep duration	0.83 (3.60)	0.82	0.62 (0.82)	0.49	-0.25 (1.33)	0.85

Abbreviations: CBT, Children with Brain Tumors; %FM, Percent Fat Mass; WHR, Waist-to-Hip Ratio; WHtR, Waist-to-Height Ratio; CI, Confidence Interval.
